# Prevalence and Clinical Characteristics of *OTOGL*-Associated Hearing Loss Identified in a Cohort of 7065 Japanese Patients with Hearing Loss

**DOI:** 10.3390/genes16020123

**Published:** 2025-01-23

**Authors:** Karuna Maekawa, Shin-ya Nishio, Kotaro Ishikawa, Masahiro Takahashi, Kozo Kumakawa, Mayuri Okami, Hidekane Yoshimura, Jun Nakayama, Masato Teraoka, Shin-ichi Usami

**Affiliations:** 1 Department of Hearing Implant Sciences, Shinshu University School of Medicine, Matsumoto 390-8621, Japan; mkarunakyuudou@me.com (K.M.); nishio@shinshu-u.ac.jp (S.-y.N.); 2 Department of Otolaryngology, National Rehabilitation Center for Persons with Disabilities, Tokorozawa 359-8555, Japan; ishikawa-kotaro@rehab.go.jp; 3 Department of Otorhinolaryngology, International University of Health and Welfare, Mita Hospital, Tokyo 108-8329, Japan; masa12_1@iuhw.ac.jp; 4 Department of Otorhinolaryngology, Akasaka Toranomon Clinic, Minato 107-0052, Japan; kozo3000@hotmail.com; 5 Department of Otorhinolaryngology, Tokai University School of Medicine, Isehara 259-1193, Japan; mayuri.okami@gmail.com; 6 Department of Otorhinolaryngology, Shinshu University School of Medicine, Matsumoto 390-8621, Japan; yoshimura@shinshu-u.ac.jp; 7 Department of Otorhinolaryngology, Shiga University of Medical Science, Otsu 520-2192, Japan; jfukui@belle.shiga-med.ac.jp; 8 Department of Otolaryngology, Head and Neck Surgery, Ehime University Graduate School of Medicine, Toon 791-0295, Japan; mteraoka@m.ehime-u.ac.jp

**Keywords:** *OTOGL*, non-syndromic hearing loss, DFNB84B, mild-to-moderate hearing loss

## Abstract

Background/Objectives: Hearing loss is one of the most common sensorineural impairments, and approximately 60% of early-onset cases are due to genetic variations. The otogelin-like protein, encoded by the *OTOGL* gene, is a component of the acellular membranes of the inner ear, such as the tectorial membrane, and is thought to play an important role in cochlear amplification. *OTOGL* gene variants are a rare cause of hearing loss such as DFNB84B, a mild-to-moderate sensorineural hearing loss presenting in early childhood with autosomal recessive inheritance. In this study, we aim to enhance our comprehension of the phenotypes of hearing loss caused by *OTOGL* variants. Methods: A total of 7056 Japanese patients with hearing loss were recruited, and based on massively parallel DNA sequencing on 158 target genes, we selected patients with biallelic *OTOGL* variants. Results: Ten affected individuals with *OTOGL* gene variants were detected, the largest group of patients yet to be reported, and eight of the eleven variants were novel. Our results showed that variations in this gene led to mild-to-moderate non-progressive hearing loss, and the accompanying symptoms, mainly vestibular symptoms, were speculated to present in adulthood. Conclusions: Determination of the phenotypes of genes causative of hearing loss is expected to greatly benefit patients with hearing loss as it can assist in predicting outcomes and lead to appropriate intervention, which, in *OTOGL*-associated hearing loss cases, is based around the fact that the patients need not be concerned with deterioration in hearing, but require careful follow-up for vestibular symptoms.

## 1. Introduction

Hearing loss (HL) is one of the most common sensory impairments, affecting approximately 1.62 in 1000 newborns [1]. In developed countries, genetic causes account for at least half of congenital or childhood-onset HL cases [1,2]. To date, variations in 89 genes have been identified as a cause of autosomal recessive non-syndromic HL (ARNSHL), which is the most common type of genetic HL (Hereditary Hearing Loss Homepage: https://hereditaryhearingloss.org). The major causative genes of non-syndromic HL in Japanese patients are *GJB2* (12.9%), *SLC26A4* (3.4%), and *CDH23* (3.4%), whereas the ratio shifts to *GJB2* (7.8%), *STRC* (3.4%), and *KCNQ4* (2.2%) among patients classified with mild-to-moderate HL [3].

The *OTOGL* gene is located on chromosome 12q21.21, which consists of 58 exons and encodes the 2344 amino acid otogelin-like protein. This protein consists of an EGF-like domain, four von Willebrand factor (vWF) domains, four cysteine-rich domains (C8), three trypsin inhibitor-like domains (TIL), an α-L-AbfB (α-L-arabinofuranosidase B) domain, and a C-terminal cystine-knot domain [4]. The otogelin-like protein has a similar amino acid sequence to the otogelin protein encoded by the *OTOG* gene, with 33.3% identity. Both proteins are non-collagenous components of the acellular membranes overlying the mechanosensory epithelia of the inner ear [5]. Otogelin and otogelin-like protein have been observed by immunofluorescence labeling in the mouse inner ear to be prominently expressed in the acellular gelatinous membranes of the cupula, the otoconical membrane, and, most prominently, in the tectorial membrane (TM), along within the Claudius cells, Hensen’s cells, and outer hair cells (OHCs) [6]. A murine study showed that the otogelin-like protein is one of the TM components and also plays a role in the horizontal top connectors and TM-attachment crowns of OHC stereocilia, anchoring the tall stereocilia of the OHCs to the TM, which are thought to enhance mechanical amplification [7].

Variations in the *OTOGL* gene are known to cause DFNB84B, a mild-to-moderate sensorineural HL presenting in early childhood with autosomal recessive inheritance [5]. Due to the limited number of reports, it has been difficult to determine the detailed clinical features for *OTOGL*-associated HL. However, recently, the detection of novel variants of these rare causative genes has been significantly simplified through the introduction of massively parallel DNA sequencing (MPS) analysis, and this has provided a clearer view of the phenotypes of gene-associated HL. The accumulation of these data has led us to a more precise prediction of patient treatment outcomes, thereby providing useful information to better assist in the decision making related to the course of treatment and/or HL intervention for affected patients.

In this study, we conducted MPS analysis for 7065 HL subjects, identified eight probands with biallelic *OTOGL* variants, and performed a retrospective analysis of the detailed clinical characteristics of 10 Japanese patients with HL (including eight probands and two affected siblings) with *OTOGL*-associated HL. From these 10 patients, we identified 11 variants, including nine novel *OTOGL* variants, detected by MPS. Through addition to the limited number of reports so far, we aim to enhance our understanding of the HL phenotypes caused by variations in the *OTOGL* gene.

## 2. Materials and Methods

### 2.1. Subjects

For this study, a total of 7065 Japanese patients with HL (autosomal dominant or maternal inheritance, 1699; autosomal recessive or sporadic, 4542; and inheritance unknown, 824) were enrolled from 102 institutions nationwide, as described previously [3]. The inclusion criteria for this genetic analysis were non-syndromic or syndromic patients with HL diagnosed in any of the 102 participating otolaryngology departments and who agreed to participate in this study. Among these subjects, we selected patients with biallelic *OTOGL* variants using MPS of 158 target genes and performed a retrospective chart review to clarify the clinical characteristics for *OTOGL*-associated HL. A detailed flowchart of the subjects enrolled in this study is shown in Supplemental Figure S1. Prior to their involvement in this study, all patients (or their guardian, caretaker, or next of kin in the case of minors or children) provided written informed consent. The Shinshu University Ethical Committee and each collaborating institution gave their approval for this study. Clinical information and peripheral blood samples were collected from each individual and their consenting relatives. This study was carried out in compliance with the Declaration of Helsinki, and the protocol was approved by the Shinshu University Ethical Committee (no. 387—4 September 2012, no. 576—2 May 2017, and no. 718—7 March 2022).

### 2.2. Variant Analysis

In this study, we used super multiplex PCR to enrich the target genome region. Sequencing libraries were prepared with an Ion AmpliSeq Custom Panel (ThermoFisher Scientific, Waltham, MA, USA) for 158 causative genes for non-syndromic or syndromic HL with the Ion AmpliSeq Library Kit 2.0 (ThermoFisher Scientific), according to the manufacturer’s procedure, which has been described elsewhere [8]. After the amplicon libraries had been prepared, sequencing was performed using the Ion S5 plus system with the Ion 540 Kit-Chef and Ion 540 Chip Kit (ThermoFisher Scientific), according to the manufacturer’s procedure.

A Torrent Mapping Alignment Program was used to align the sequence data against the human genome sequence (build GRCh37/hg19). Following sequence mapping, the DNA variants were picked up using the Torrent Variant Caller plug-in program ver. 5.16. The repercussions of detected variants were assessed using the ANNOVAR software ver. 2020-06-08 [9]. The detected variants were filtered based on the following criteria: (1) protein-affecting variants (including the missense, nonsense, insertion/deletion, and splicing variants) and (2) an allele frequency of less than 1% in several control databases (including the 1000 genome database [10], The Genome Aggregation Database [11], the human genetic variation database (dataset for 1208 Japanese exome variants) [12], the 54,000 Japanese genome variation database (ToMMo 54KJPN) [13], and the 333 in-house Japanese controls with normal hearing). Filtering was performed with the original database software described in our previous paper [14]. Sanger sequencing was used to validate the identified candidate variants. We also performed copy number analysis using the read depth data for all 158 genes obtained from the NGS analysis, according to the methods in our previous paper [15].

The pathogenicity of the identified variants was evaluated according to the American College of Medical Genetics (ACMG) standards and guidelines [16], with the ClinGen HL clinical domain working group expert specification [17]. Candidate variants were selected among the identified variants based on the following criteria: (1) variants previously reported as “pathogenic” or “likely pathogenic” without any conflicting evidence, (2) novel variants classified as “pathogenic” or “likely pathogenic”, and (3) variants of “uncertain significance” (VUS) identified as the only candidate after the filtering procedure, without any candidate variants in the other 157 genes.

### 2.3. Clinical Evaluations

We collected data regarding the onset age, gender, progressiveness of HL, pedigree, and episodes of vertigo. Evaluation of HL was performed using pure-tone audiometry on patients aged 5 years or older, and auditory steady state response (ASSR), conditioned orientation response audiometry (COR), or play audiometry on patients under 5 years old. Audiometric thresholds at four frequencies (0.5, 1, 2, and 4 kHz) were used to calculate the pure-tone average (PTA). The severity of HL was classified into four categories: mild (PTA > 25 dB and ≤40 dB HL), moderate (>40 dB and ≤70 dB HL), severe (>70 dB and ≤90 dB HL), and profound (>90 dB). The audiometric configurations were classified as flat, low-frequency HL, mid-frequency HL, sloping high-frequency HL (gradually worsened 10 dB for high frequency), or precipitous high-frequency HL (higher frequency thresholds deteriorated by at least 20 dB per octave).

## 3. Results

### 3.1. Identified OTOGL Variants

We identified 11 possibly disease-causing *OTOGL* variants, of which eight were novel (Table 1). The novel variants consisted of one missense variant, two splicing variants, three nonsense variants, and two frameshift deletion variants. The minor allele frequency of all variants was less than 0.07% based on the aforementioned database, fulfilling the criteria supporting variant pathogenicity as proposed by the ClinGen HL expert panel. Based on the ACMG guidelines, five of these novel variants were categorized as likely pathogenic variants and three as variants of uncertain significance. The low carrier frequencies in the Japanese control population database (ToMMo 54KJPN) also support the pathogenicity of these variants. We could not conduct a segregation analysis in some families, but no other biallelic recessive gene variations were detected in our filtering of the MPS results.

### 3.2. Prevalence and Clinical Characteristics of OTOGL-Associated HL

**Figure 1:**
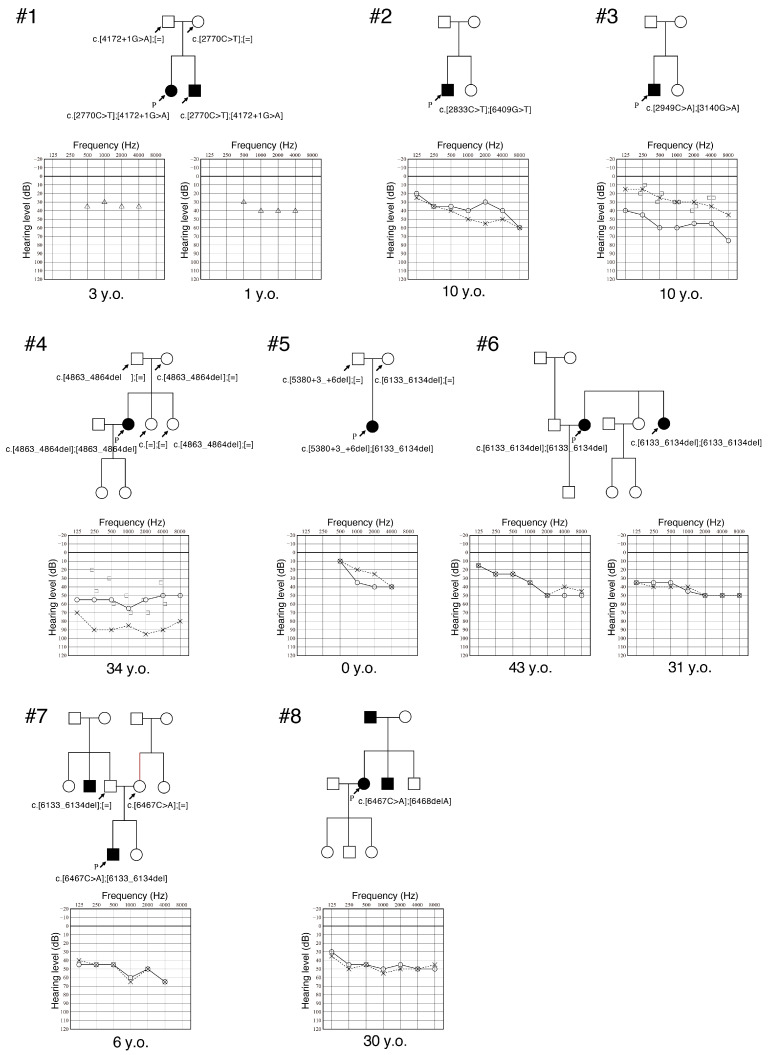
Pedigree and audiograms for the family of each of the patients with *OTOGL*-associated HL identified in this study. The variants identified in this study are indicated in the figure. Solid line: hearing threshold in the right ear; Dashed line: hearing threshold in the left ear.

In this study, we identified ten affected individuals from eight families with biallelic *OTOGL* variants (Figure 1 and Table 2). *OTOGL* is a relatively rare causative gene, and the prevalence of *OTOGL*-associated HL is 0.11% (8/7065) among Japanese patients with HL and 0.18% (8/4542) among autosomal recessive or sporadic HL cases.

The clinical features are summarized in Table 2. The onset age of HL was congenital in four cases and within their first decade of life for the other six cases. Four patients had mild HL and six had moderate HL. Patient #3 was diagnosed with otitis media with effusion in his right ear when we obtained his audiogram, explaining the air–bone gap. Patient #4 had initially been diagnosed with otosclerosis, but this was later proven otherwise when normal stapes mobility was observed upon surgery undertaken when she was 13 years old. The configuration of their audiograms was flat-type HL (6/10) or sloping high-frequency gently sloping-type HL (4/10). Four patients were aware of HL progression, and three of them complained of vestibular symptoms. Patients from family #4 and #6 also noticed fluctuation in their HL accompanying its progression.

## 4. Discussion

**Figure 2:**
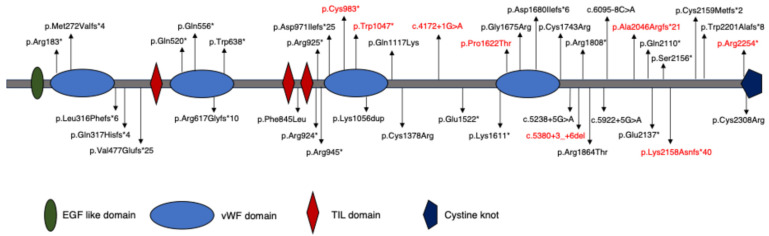
All pathogenic *OTOGL* variants that have been reported and their locations in the *OTOGL* gene. The variants in red letters are the novel variants identified in this report. *: stop codon.

In this study, we identified eight novel *OTOGL* variants by MPS among ten Japanese HL patients identified to have *OTOGL* variants. To date, 36 *OTOGL* variants have been reported from around the world, consisting of 21 individual cases, making this report the largest group of patients to date (Table 3). Apart from one variant, all variants detected in this report were truncating variations (88.9%), which is similar to the ratio observed in previous studies (86.1%, Table 3). We did not find any correlation between the location of the *OTOGL* variants and the phenotype (Figure 2). Our results indicate that the underlying pathogenic mechanism for *OTOGL*-associated HL is the loss of otogelin-like protein function.

Our results showed that all patients with *OTOGL* variants presented congenital or early-onset mild-to-moderate HL. Most previous cases also showed congenital or early-onset mild-to-moderate HL, which is consistent with our results (Table 3). The otogelin-like protein is mainly localized in the tip of OHCs stereocilia and the TM and plays a crucial role in cochlear amplification. A loss of distortion product otoacoustic emission (DPOAE), which reflects deficit in OHC function, has been observed in patients with *OTOGL*-associated HL [4,7]. As seen in previous reports, most of the genes associated with cochlear amplification, including *OTOG*, *OTOGL*, *STRC*, *TECTA,* and *OTOA* are related to mild-to-moderate HL, which also supports the pathogenic mechanism for *OTOGL*-associated HL [21,22,23,24,25,26,27,28,29].

**Figure 3:**
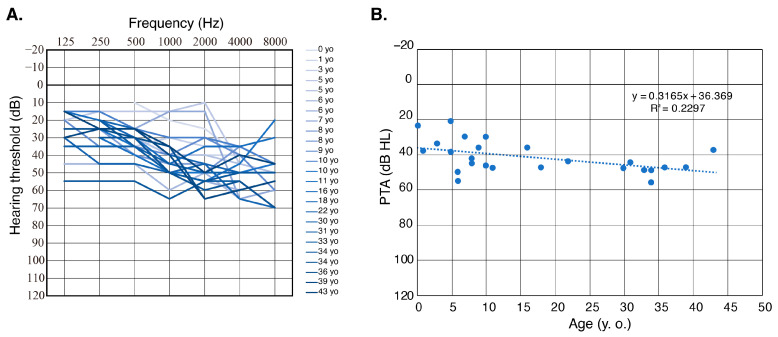
(**A**): Overlapping audiograms from this report and all previous reported cases. Ages ranged from 0 to 36 yo (mean 14.9 yo), with older patients shown in darker colors. (**B**): Detailed progression analysis of hearing deterioration for patients with *OTOGL*-associated HL. Each dot indicates the pure-tone average (PTA; average of hearing thresholds for 500 Hz, 1000 Hz, 2000 Hz, and 4000 Hz) and age of each patient. Dotted line indicates the linear regression.

Four of the ten patients (40%) in this study were aware of their HL progression, although we were not able to acquire serial audiograms from these four patients. As this did not align with previous reports, we assessed the audiograms that we were able to obtain. The one patient (family #1) from whom we were able to obtain serial audiograms did not show HL progression. In addition, we evaluated the audiograms of patients from this report and all previous reports (Figure 3A), showing that it is likely that variations in *OTOGL* do not result in clear HL deterioration. Detailed hearing deterioration analysis using scatter plotting also indicated relatively stable hearing threshold changes compared to other forms of genetically caused HL with a progressive nature (Figure 3B). In a past murine study, *OTOGL* was transcribed in high levels during the embryonic stage, after which the transcription decreased in the early postnatal stage, before decreasing even further in the adult stages. Indeed, immunostaining was most prominent in p0 mice and gradually decreased and localized mainly in the TM with development [5]. In addition, all previously reported *OTOGL*-associated HL cases showed non-progressive HL (Table 3). Thus, non-progressive HL is thought to be one of the clinical characteristics of *OTOGL*-associated HL, but further research is needed to confirm progression.

**Table 3:** Clinical characteristics of patients with *OTOGL*-associated HL in previous reports.

Nucleotide Change	AA Change	Onset	Severity	Configuration	Progression	Vestibular Dysfunction	Population	Reference
c.[547C>T];[5238+5G>A]	p.[Arg183*];[IVS43 ds G-A +5]	Congenital	Moderate	-	No	No	Dutch	[5]
c.[547C>T];[5992+5G>A]	p.[Arg183*];[IVS48 ds G-A +5]	Congenital	Mild to moderate	-	-	No	US	[30]
c.[814_815delAT];[814_815delAT]	p.[Met272Valalfs*4];[Met272Valfs*4]	Congenital	Moderate to severe	-	No	-	French-Canadian	[31]
c.[814_815delAT];[5023G>A]	p.[Met272Valfs*4];[Gly1675Arg]	Congenital	Moderate	-	No	-	French-Canadian	[31]
c.[948delG];[948delG]	p.[Leu316Phefs*6];[Leu316Phefs*6]	Congenital	Moderate	-	-	-	Ashkenazi Jewish	[32]
c.[951_954delGTTT];[6328C>T]	p.[Gln317Hisfs*4];[Gln2110*]	Perilingual	Mild	-	No	No	Korea	[33]
c.[1430delT];[4132T>C]	p.[Val477Glufs*25];[Cys1378Arg]	Congenital	Moderate	-	No	Hypofunction	Dutch	[5]
c.[1558C>T];[2773C>T]	p.[Gln520*];[Arg925*]	5 yo	Mild to moderate	Flat	No	No	France	[4]
c.[1558C>T];[4032_4054+30del53]	p.[Gln520*];[His1344Glnfs*13]	Congenital	Moderate	Mid frequency	No	No	Slovakia	[34]
c.[1666C>T];[5422C>T]	p.[Gln556*];[Arg1808*]	Congenital	Moderate	-	-	-		[35]
c.[1849delA];[6601_6602delTG]	p.[Arg617Glyfs*10];[Trp2201Alafs*8]	Congenital	-	-	-	No	US	[30]
c.[1913G>A];[1913G>A]	p.[Trp638*][Trp638*]	Prelingual	Moderate	Low frequency	No	-	Brazil	[36]
c.[2533T>C];[4833G>A]	p.[Phe845Leu];[Lys1611*]	Congenital	Severe to profound	-	No	Left hypofunction	Netherlands	[37]
c.[2770C>T];[5038delG]	p.[Arg924*];[Asp1680Ilefs*6]	4 yo	Mild	-	No	No	China	[18]
c.[2833C>T];[2911delG]	p.[Arg945*];[Asp971Ilefs*25]	Prelingual	Mild or moderate	-	-	-	China	[19]
c.[3166_3168dupAAA];[4564G>T]	p.[Lys1056dup];[Glu1522*]	Congenital	-	-	-	-	US	[30]
c.[3349C>A];[6095-8C>A]	p.[Gln1117Lys];[IVS49 as C-A -8]	Postnatal	Moderate	-	No	-		[38]
c.[4252+1G>A];[6922T>C]	p.[IVS35 ds G-A +1];[Cys308Arg]	5 yo	Mild	Down sloping	-	-	China	[39]
c.[5227T>C];[5227T>C]	p.[Cys1743Arg];[Cys1743Arg]	Prelingual	Mild to severe	-	-	-	US	[30]
c.[6467C>A];[6474dupA]	p.[Ser2156*];[Cys2159Metfs*2]	7 yo and 9 yo	Moderate	HF_gentle	No	No	China	[20]

AA: amino acid; AR: autosomal recessive; AD: autosomal dominant; M: male; F: female; HF_gentle: sloping high-frequency HL; Y: yes; N: no; and *: stop codon.

Among the four adult patients, three have experienced episodes of vestibular symptoms. Most of the cases in previous reports did not have any vestibular symptoms, but vestibular hypofunction was observed in some patients in our study [5,37]. As *OTOGL* is also expressed in the otoconial membrane, it is possible that its mutation causes vestibular dysfunction. Detachment of both the otoconial membrane and cupula was observed in *OTOG* knockout mice and this has been suggested to be the cause of impaired vestibular function [40]. We speculate that *OTOGL* may play an essential role in normal vestibular function, but further investigation into this associated symptom is required.

## 5. Conclusions

In conclusion, *OTOGL* deficiency leads to congenital or early-onset, mild-to-moderate non-progressive HL. These relatively mild forms of HL can be underestimated, especially when not detected in newborn hearing screening, and may lead to delays in speech and social development. Determination of the cause of HL in its early stage enables appropriate intervention, especially for patients in whom HL is caused by pathological gene variants that have a known phenotype. Further research is needed to investigate the correlation between *OTOGL* deficiency and vestibular symptoms, as our findings suggest that these accompanying symptoms present in adulthood in patients.

## Figures and Tables

**Table 1:** *OTOGL* variants identified in this study.

Nucleotide Change	AA Change	Exon	SIFT	PP2	LRT	MutTaster	MutAssessor	REVEL	CADD	ToMMo 54KJPN	gnomAD All	Pathogenicity	Reference
c.2770C>T	p.Arg924*	exon25	.	.	.	A	.	.	44	.	1.29 × 10^−5^	Likely Pathogenic	[18]
c.2833C>T	p.Arg945*	exon25	.	.	.	A	.	.	38	0.000287	6.42 × 10^−5^	Likely Pathogenic	[19]
c.2949C>A	p.Cys983*	exon26	.	.	.	A	.	.	37	.	.	Likely Pathogenic	This study
c.3140G>A	p.Trp1047*	exon27	.	.	.	A	.	.	43	.	.	Likely Pathogenic	This study
c.4172+1G>A		exon34	.	.	.	D	.	.	26.6	.	.	Likely Pathogenic	This study
c.4863_4864delinsAA	p.Pro1622Thr	exon42	.	.	.	.	.	.	.	.	.	VUS	This study
c.5380+3_+6del		exon49	.	.	.	.	.	.	.	9.2 × 10^−5^	2.728 × 10^−5^	VUS	This study
c.6133_6134del	p.Ala2046Argfs*21	exon50	.	.	.	.	.	.	.	0.000184	1.613 × 10^−5^	Likely Pathogenic	This study
c.6409G>T	p.Glu2137*	exon53	.	.	N	A	.	.	53	0.000166	1.413 × 10^−5^	Likely Pathogenic	This study
c.6467C>A	p.Ser2156*	exon53	.	.	D	A	.	.	55	0.000267	0.0001	VUS	[20]
c.6468delA	p.Lys2158Asnfs*40	exon53	.	.	.	.	.	.	.	4.6 × 10^−5^	4.367 × 10^−5^	Likely Pathogenic	This study

All variants are indicated on NM_173591. AA: amino acid; PP2: PolyPhen2; MutTaster: mutation taster; MutAssessor: mutation assessor; VUS: variant of uncertain significance; and *: stop codon.

**Table 2:** Clinical characteristics of patients with *OTOGL*-associated HL identified in this study.

Family Number	ID	Base Change Allele 1	AA Change Allele 1	Base Change Allele 2	AA Change Allele 2	Hereditary	Onset	Age	Gender	Severity of HL	Type of HL	Progression	Vestibular Symptoms
1	JHLB-0872	c.2770C>T	p.Arg924*	c.4172+1G>A	Spl.	AR	0	3	F	Mild	Flat	N	N
	JHLB-2210	c.2770C>T	p. Arg 924*	c.4172+1G>A	Spl.	AR	0	1	M	Mild	Flat	Y	N
2	JHLB-1937	c.2833C>T	p. Arg 945*	c.6409G>T	p.Glu2137*	Sporadic	0	10	M	Moderate	HF_gentle	N	N
3	JHLB-7125	c.2949C>A	p.Cys983*	c.3140G>A	p.Trp1047*	Sporadic	6	10	M	Moderate	HF_gentle	N	N
4	JHLB-3844	c.4863_4864delinsAA	p.Pro1622Thr	c.4863_4864delinsAA	p.Pro1622Thr	Sporadic	5	34	F	Moderate	Flat	Y	Y
5	JHLB-7091	c.5380+3_+6del	Spl.	c.6133_6134del	p.Ala2046Argfs*21	Sporadic	0	0	F	Mild	HF_gentle	N	N
6	JHLB-6995	c.6133_6134del	p.Ala2046Argfs*21	c.6133_6134del	p.Ala2046Argfs*21	AR	6	43	F	Mild	HF_gentle	Y	Y
	JHLB-6996	c.6133_6134del	p.Ala2046Argfs*21	c.6133_6134del	p.Ala2046Argfs*21	AR	9	31	F	Moderate	Flat	N	N
7	JHLB-10297	c.6133_6134del	p.Ala2046Argfs*21	c.6467C>A	p.Ser2156*	Sporadic	4	6	M	Moderate	Flat	N	N
8	JHLB-0223	c.6467C>A	p.Ser2156*	c.6468delA	p.Lys2158Asnfs*40	AD	10	30	F	Moderate	Flat	Y	Y

AA: amino acid; AR: autosomal recessive; AD: autosomal dominant; M: male; F: female; HF_gentle: sloping high-frequency HL; Y: yes; N: no; and *: stop codon.

## Data Availability

The datasets used during the current study are available from the corresponding author upon reasonable request.

## Supplementary Materials

The following supporting information can be downloaded at: https://www.mdpi.com/article/10.3390/genes16020123/s1, Figure S1: Detailed flowchart of the subjects enrolled in this study.
