# Supplementary material for: Prevalence and Clinical Characteristics of OTOGL-Associated Hearing Loss Identified in a Cohort of 7065 Japanese Patients with Hearing Loss
Source: Genes (Basel). 2025 Jan 23;16(2):123. doi: 10.3390/genes16020123 (PMC11854685; doi:10.3390/genes16020123)
Supplement: Supplementary file 1 [file genes-16-00123-s001.zip › genes-3421235-supplementary.pdf]

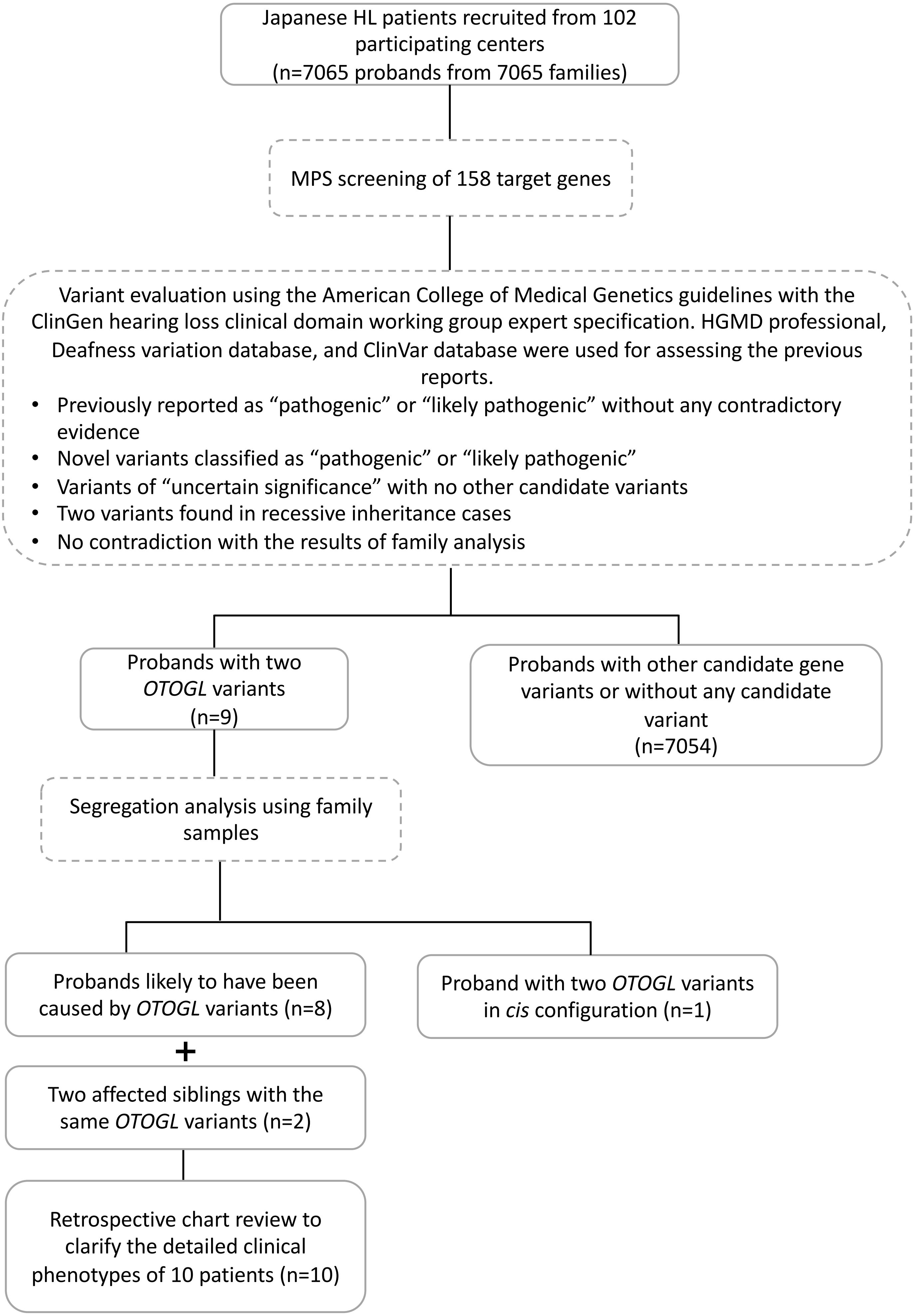
Japanese HL patients recruited from 102 participating centers (n=7065 probands from 7065 families)
MPS screening of 158 target genes
Variant evaluation using the American College of Medical Genetics guidelines with the ClinGen hearing loss clinical domain working group expert specification. HGMD professional, Deafness variation database, and ClinVar database were used for assessing the previous reports.
• Previously reported as “pathogenic” or “likely pathogenic” without any contradictory evidence
• Novel variants classified as “pathogenic” or “likely pathogenic”
• Variants of “uncertain significance” with no other candidate variants
• Two variants found in recessive inheritance cases
• No contradiction with the results of family analysis
Probands with two *OTOGL* variants (n=9)
Probands with other candidate gene variants or without any candidate variant (n=7054)
Segregation analysis using family samples
Probands likely to have been caused by *OTOGL* variants (n=8)
Proband with two *OTOGL* variants in *cis* configuration (n=1)
+
Two affected siblings with the same *OTOGL* variants (n=2)
Retrospective chart review to clarify the detailed clinical phenotypes of 10 patients (n=10)
